# Successful Strategy in Creating Low-FODMAP Wholegrain Bread—Simple and Global

**DOI:** 10.3390/foods14020304

**Published:** 2025-01-17

**Authors:** Aleksandra M. Torbica, Vesna Vujasinović, Uroš Miljić, Goran Radivojević, Bojana Filipčev, Milorad Miljić, Miloš Radosavljević

**Affiliations:** 1 Institute of Food Technology, University of Novi Sad, Bulevar cara Lazara 1, 21102 Novi Sad, Serbia; bojana.filipcev@fins.uns.ac.rs (B.F.); milorad.miljic@fins.uns.ac.rs (M.M.); 2 Faculty of Sciences, University of Novi Sad, Trg Dositeja Obradovića 3, 21000 Novi Sad, Serbia; vesna.vujasinovic@dgt.uns.ac.rs (V.V.); goran.radivojevic@dgt.uns.ac.rs (G.R.); 3 Faculty of Technology, University of Novi Sad, Bulevar cara Lazara 1, 21102 Novi Sad, Serbia; urosch@uns.ac.rs (U.M.); milosr@tf.uns.ac.rs (M.R.)

**Keywords:** fructo-oligosaccharides, galacto-oligosaccharides, wholegrain flours, FODMAP, baker’s yeast, breadmaking

## Abstract

Fermentable oligo-, di-, and monosaccharides as well as polyols (FODMAPs) came into focus following recent clinical studies confirming that they worsen the symptoms of several gastrointestinal disorders suffered by 40% of the general population. Currently; only the low-FODMAP diet is a valuable strategy to help relieve IBS symptoms; however; it is only a temporary solution due to the nutritional deficiency caused by avoiding high-FODMAP foods. At the same time; bakery products are an important part of the human diet worldwide and the key contributors to the high intake of FODMAPs; especially in their wholegrain form. Previous research has shown that reducing FODMAPs content has negative effects on the structures of dough and bread; as well as on sensory quality. Our innovative low-FODMAP wholegrain bakery products provide a unique solution for achieving a high-dietary-fiber intake without compromising the sensory appeal. The novelty of our work is that these experiments were the first to be performed based on known but unexploited facts about the superiority of the baker’s yeast enzymatic complex. A crucial reduction in FODMAP content (by more than 75%) was achieved via a simple alteration to the bread formulation (6% baker’s yeast and the addition of baking powder) and key process parameter values (40 °C and 60 min dough fermentation time) in conventional breadmaking technology.

## 1. Introduction

In recent years, the human diet has become the center of both a healthy lifestyle and therapies for people suffering from gastrointestinal problems. Functional gastrointestinal disorders (FGIDs) represent some of the most common gastrointestinal illnesses and reported causes for sick leave and extended medical treatments. Irritable bowel syndrome (IBS) is one of the most noticeable FGIDs, with a prevalence of 15–20% worldwide and, significantly, 8% among the European population [1]. Although the general guidelines for IBS conclude that “fiber provides overall symptom relief in IBS”, advancements in technology and analytical tools are providing new evidence of the dual nature and effect of DF on the subject at hand. When isolated fibers were assessed in randomized, placebo-controlled clinical studies, the results suggested that fibers do not have a beneficial effect on diarrhea, chronic idiopathic constipation (CIC), and IBS. Furthermore, emerging clinical evidence is generally inconclusive and further investigation is needed [2]. However, over the last two decades (especially in the last decade) the negative effect of one aspect of DF has been proven through clinical trials. FODMAPs (Fermentable oligo-, di-, and monosaccharides and polyols) are dietary fiber compounds newly suspected to trigger IBS and several associated gastrointestinal disorders [3,4,5]. FODMAP compounds include fructans, fructo-oligosaccharides and galacto-oligosaccharides, galactans, lactose, fructose when in excess to glucose, sorbitol, mannitol, maltitol, and xylitol (sugar alcohols) [6]. A diet low in FODMAPs reduces gastrointestinal symptoms in patients with irritable bowel syndrome (IBS), with a symptomatic response rate of 50–70% demonstrated across multiple randomized controlled trials. However, this dietary approach of excluding a significant number of ingredients and food may potentially reduce fiber intake, which might impact general gut health and exacerbate constipation, as well as alter the colonic microbiota and luminal fermentation [7,8]. Considering the daily intakes and portions of different ingredients in the daily diet, wholegrain cereals and pulses, as well as derivative products, are a considerable source of energy, dietary fiber, and micronutrients necessary for a healthy diet [9]. Furthermore, wheat and wheat-based products account for one-fourth of the daily calorie intake in the European population, and a significant proportion of these products are bakery products designated as staple foods. However, since cereals like wheat and rye (which primarily contain fructans and FOS) and pulses (which are rich in GOS) are natural source of FODMAPs, IBS patients must largely avoid products that are made from them [10,11,12,13]. The best solution for IBS patients is the production and global availability of high-quality, sensory acceptable, and nutritious low-FODMAP products that can replace existing conventional high-FODMAP products.

The dietary fiber (DF) content is modified and the FODMAP compound content is reduced in bakery products via several biotechnological processes. These processes include the addition of enzymes (endogenous and exogenous) and microbial fermentation (in the form of yeast and/or sourdough fermentation) as part of the standard or modified bread-making process. The use of naturally occurring processes, microbiota, and enzymes not only benefits the general population but also decreases the chemical burden on the environment [14]. Yeast fermentation in bread production is the easiest to implement under the current food industry conditions, and the commonly used biological leavening agent in present-day bakeries is *Saccharomyces cerevisiae* (baker’s yeast) [15].

During breadmaking, endogenous, microbial, and added commercial enzymes (amylases and other carbohydrate hydrolyzing enzymes) break down starch to fermentable sugars such as maltose. Subsequently, dough is then leavened via bacterial and/or yeast fermentation, and the sugars are converted (partially) to carbon dioxide to achieve increased volume and fluffiness. The structure of wheat dough is definitively affected by *S. cerevisiae* (as the leavening agent) and its metabolic products (carbon dioxide, organic acids, and probably glutathione) [15]. The dough matrix is strongly influenced by products of yeast metabolism, and the rheological characterization of the dough has presented a challenge in several studies due to extreme sensitivity of the tests. Furthermore, the yeast concentration and fermentation time are decisive factors decreasing the pH of dough via the production and accumulation of acidic reagents. The authors assumed that the decrease in pH during dough fermentation positively affected the protease activity, which resulted in a weakening of the gluten structure. The pH value and fermentation time are therefore responsible for the constant decrease in stiffness of dough through the enzymatic cleavage of the gluten network. The optimum pH of proteases is around 4.4 [16], and this information must be carefully considered regarding the optimization of the breadmaking process to minimize gluten network weakening and enable the formation of an appealing bread loaf.

In recent years, a series of studies have assessed fructans degradation via yeast, as well as its accompanying challenges. Although baker’s yeast degrades fructans during fermentation, conventional wholegrain wheat breads often retain a high FODMAP compounds content. In a study investigating 96 yeast isolates from different environments, only 2 promising isolates for FODMAPs reduction were identified: *Lachancea fermentati* FST 5.1 and *Cyberlindnera fabianii* NTCyb. Their potential to produce low-FODMAP wholegrain wheat bread was compared to *S. cerevisiae.* The yeast strain *Lachancea fermentati* FST 5.1 was proposed as an alternative to baker’s yeast since it produces low-FODMAP wholegrain wheat bread while sustaining an optimal bread quality and consumer acceptance [17]. However, the time for the fermentation and proofing of dough was increased to 150 min in total (90 + 60 min). Ziegler et al. [18] have determined that a sufficiently long proofing time is much more important than the selection of a low-fructan wheat grain type for yeast-mediated FODMAP reduction. In their research, after 60 min of fermentation and proofing, a fructan degradation of 60% was achieved; however, the fructose content released from raffinose, sucrose, and fructans reached a level of around 1% excess fructose in the bread. Extending the fermentation time to 2.5 h facilitated a reduction in the excess fructose by ~70% and led to low FODMAP levels overall. Furthermore, fermentation and proofing for 4.5 h resulted in >90% fructan degradation and only 0.03% excess fructose. However, in industrial breadmaking, fermentation times are often too short to achieve sufficiently low FODMAP levels, especially in wholegrain breads, which presents a significant technological challenge [19].

Understanding baker’s yeast metabolism is a crucial aspect in developing a FODMAP-reduction strategy in bread. Analyzing the current literature on *S. cerevisiae* and its specific enzymes, optimal pH and temperature values play vital roles in FODMAP degradation. *S cerevisiae* possesses invertase [20], which has a higher affinity towards short-chain fructans and quickly degrades fructans with a degree of polymerization (DP) of up to five in the first hour of fermentation. However, the degradation of fructans with a higher DP is slower [13]. According to Struyf et al. [21], yeast invertase can degrade fructo-oligosaccharides with a DP > 4, and can also degrade branched fructan structures, for example, branched graminan- and neo-type fructans present in wheat. During the first 60 min of fermentation, the remaining tri- and tetrasaccharides were mostly hydrolyzed, and the pentasaccharide content was reduced to about 50%. Additionally, 30% of fructans with a higher DP had been hydrolyzed during this time. During the following 40 min only, a small amount was further degraded [22]. The optimal pH for invertase activity is in the range of 4.5 to 6.5 for all substrates except inulin, which exhibits an optimum activity at pH 6.0, while the optimal temperature is in range of 30–55 °C [23,24,25,26]. The literature data from a large number of reports suggest that the optimum pH and temperature for fructosyltransferase activity is between 4.5 and 6.5 and 40–60 °C, respectively [23]. The third crucial enzyme, fructose-bisphosphate aldolase, often called aldolase, is a glycolytic enzyme that catalyzes the conversion of fructose 1-6-diphosphate to glyceraldehyde 3-phosphate and dihydroxy-acetone phosphate via the glycolysis metabolic pathway [27]. Yeast aldolase is a highly negatively charged molecule at pH 7.5 [28] with optimum pH values of 7.0 [29]. Yeast α-galactosidases, responsible for GOS reduction, typically have pH optima in the range of 3.5–5.0 [15]. Although inulin is not present in wheat flour, the inulinase present in bakers’ yeast might be of importance for the hydrolysis of wheat fructans, since some of the cereal fructans have been reported to have an ’inulin type’ structure, specifically chains with (2→I)-fructofuranosidic linkages between the fructose units [22].

The efficiency of FODMAP compound degradation during fermentation via baker’s yeast has been evaluated positively in only two studies [17,18]. Other studies agree that during the first 60 min of fermentation, there is a significant reduction in fructan, but that degradation products simultaneously accumulate and slow down further degradation of fructan [30].

In an experiment, whereby an overdose of commercial *S. cerevisiae* block yeast (16% on wholegrain basis) was used, 93% of the fructans present in wholegrain wheat were degraded during dough fermentation (after 2 h), suggesting that at least 93% of the wheat fructans were transported through the yeast cell wall. These high dosages would, however, result in excessive CO_2_ production during proofing and the collapse of the dough structure, and might also lead to off-flavor formation [21].

Due to the evident shortcomings of yeast fermentation in dough, the sourdough process has become the only feasible technological process to reduce FODMAPs in breadmaking. Recent research has shown that fructans and raffinose contents decrease via sourdough fermentation, with yeast having a greater effect, and an increase in organic acids and mannitol has also been observed. Yeast breads have higher total sugar, polyol, and organic acid contents than sourdough and mixed breads [31]. In sourdough fermentation, yeasts transform hexoses into ethanol and CO_2_, while sourdough LABs transform hexoses into lactic acid. However, sourdough fermentation is not cost effective in comparison to yeast fermentation on an industrial scale, and it yields breads with different sensory profiles in comparison to yeast breads.

Besides the health aspect, sensory characteristics and overall appeal play decisive roles in consumers’ selection of breads. Volatile bread compounds include alcohol, organic acid, aldehyde, terpene, and ester compounds. Besides the baking process, which mostly influences the typical aroma of the bread crust, a crucial step for developing the flavor of the crumb is dough fermentation. Among the diverse variations in this process, the type of leavening agent applied has a significant impact on flavor and aroma [31]. Considering the factors detailed above, the selection of leavening agents, the duration of the fermentation, and the specific choice of additives determine and facilitate the production of breads with distinctive and appealing sensory profiles.

Furthermore, the addition of sources of non-FODMAP fibers is beneficial for the enhancement of the nutritional value of low-FODMAP foods, contributing to an increase in total dietary fibers. In clinical placebo-controlled studies, psyllium, classified as nonfermented gel-forming fiber, has been shown to improve bowel function for several diarrhea and incontinence disorders, and has a positive effect on ulcerative colitis, IBS, colon cancer, diabetes, and hypercholesterolemia [8,32]. Chia seeds have also been shown to be an important source of dietary fiber (both soluble and insoluble), proteins, omega-3 fatty acids, and bioactive and polyphenolic compounds, thus making them highly suitable for the food industry. Chia seeds serve as a good thickener, gel-forming and chelating agent, foam enhancer, emulsifier, suspending agent, and rehydration agent. The beneficial effects of chia seeds have also been observed for health problems such as dyslipidemia, inflammation, insulin resistance, and cardiovascular diseases [33].

**Figure 1:**
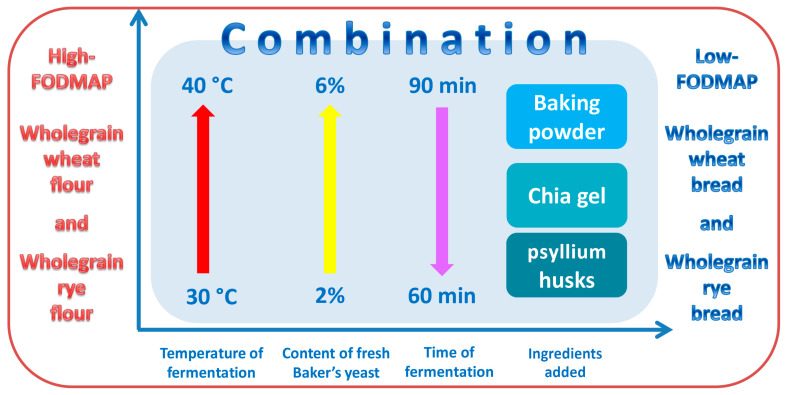
Study design.

The development of low-FODMAP products and the nutritional enhancement of corresponding products is of utmost importance, as supported by the above overview of the currently limited research in the field of FODMAPs and by the initial findings supporting evidence for the adverse effects of FODMAPs regarding health and the induction or the worsening of functional gastrointestinal disorders. The current research on the creation of low-FODMAP breads is still in its early stage, addressing only one or a few process parameters of breadmaking without detailed optimization. An overall approach to the creation of low-FODMAP breads is needed, wherein the focus must not only be on the reduction in FODMAP compounds but also on the cost efficiency of the process and the sensory characteristics and general appeal of the products. In this study, the crucial steps for optimizing the breadmaking process (and managing the subsequent effects) include managing the temperature and duration of fermentation, dough pH control via the addition of chemical leavening agents (baking powder), establishing the concentration of biological leavening agents (baker’s yeast), and the addition of fiber-enhancing additives (chia and psyllium). The effects of these optimization procedures on bread structure, chemical composition, and overall sensory acceptance were investigated, with the final goal of achieving an optimized, industry-appropriate process for the production of low-FODMAP breads with a high fiber content (Figure 1).

## 2. Materials and Methods

### 2.1. Material

The wheat wholegrain flour, rye wholegrain flour, baker’s yeast, salt (NaCl), sunflower oil, baking powder, sucrose, ground chia seeds, and ground psyllium husk used in this study were acquired from a local market.

### 2.2. Water Absorption

The optimal water absorption of wholegrain wheat and rye flour was determined using the Perten Instruments Method performed on Micro-doughLAb (Perten Instruments, Stockholm, Sweden) at a mixing speed of 120 rpm for 10 min by targeting a peak resistance of 500 FU at a temperature of 30 °C (Micro-doughLAB 2800 Installation and Operation Manual, 2014) [34,35].

### 2.3. Breadmaking Procedure

For our analysis and sensory evaluation, breads were prepared according to the procedures shown in Table 1.

All ingredients (according to each column in Table 1) were mixed at a low speed (85 rpm/min) in a mixer for 3–5 min, followed by mixing at 100 rpm/min for 2 min in a table mixer (CONTI Planetary table Mixer mod. PL11 8B, Bussolengo, Verona, Italy).

Ground chia seeds were mixed with the remaining water (total water needed for dough preparation minus the water for yeast activation) and left for 30 min for the chia seeds to swell and form a gel-like structure. The grounded psyllium husk was added to the wholegrain flours dry.

Control samples were produced using standard breadmaking conditions (90 min fermentation, 30 min leavening at 30 °C) with 2% baker’s yeast, while the other samples were produced using 6% baker’s yeast and the specific designed conditions. After kneading, each sample (including the controls) was divided into two equal portions, and these were left to rest for 90 or 60 min, at 30 or 40 °C and 90% relative humidity in a proofing chamber (BONGARD, Holtzheim, France), according to the procedures in Table 1. Samples were then gently molded into cylindrical shapes, put into stainless steel pans (length: 9 cm; width: 7.5 cm; height: 5.5 cm), and leavened at 30 or 40 °C and 90% relative humidity in a proofing chamber (BONGARD, Holtzheim, France) for 30 min (according to the procedures in Table 1). All the leavened dough samples were then baked (Mac-Pan^®^, Thiene, Italy) at 180 °C for 30–35 min. Two loaves were yielded for each sample in each baking test.

### 2.4. Quantification of FODMAPs Using the HPAEC-PAD Method

All samples were freeze-dried and then ground. Samples were frozen and stored at −30 °C for 24 h and then subjected to lyophilization using a ChristALPHA1-2 LDPLUS device (Osterode am Harz, Germany). The lyophilization parameters were set as follows: the main drying process was performed at 0.01 bar for 24 h at a condenser temperature of −40 °C and a shelf temperature of 20–30 °C (room temperature), and the final drying lasted for 24 h at 0.005 mbar at a condenser temperature of −57 °C and a shelf temperature of 20–30 °C (room temperature). An amount of 50 mL of ultrapure water was added to 0.5 g of ground sample, and subsequently the samples were placed in a boiling water bath for 10 min. The samples were then cooled to room temperature and centrifuged at 8000 rpm at 4 °C for 10 min. After centrifugation, the separated supernatant was frozen at −20 °C (to make it easier to separate the sample from any fat present and to avoid the use of organic solvents). After thawing, the frozen supernatant was again centrifuged at 4 °C for 5 min at a speed of 8000 rpm. Finally, 5 mL of the sample thus prepared was transferred to vials for HPAEC-PAD.

Targeted carbohydrates were separated and quantified using a Dionex ICS-6000+ system (Sunnivale, CA, USA) and an electrochemical detector (ED) with a gold working electrode and a AgCl reference electrode.

For monosaccharides, disaccharides, and polyols, the eluents were 10 mM NaOH, 200 mM NaOH, and ultrapure water. Monosaccharides, disaccharides, and polyols were separated on a Thermo Scientific Dionex CarboPac PA20 analytical column (3 × 150 mm) (Thermo Scientific, Sunnyvale, CA, USA) with an appropriate protective column using isocratic elution, according to the method of Weitzhandler et al. [36].

Oligosaccharides (FOS and GOS) were separated using a Thermo Scientific Dionex CarboPac PA200 analytical column (3 × 250 mm), with an appropriate guard column, using gradient elution according to the method of Ispiryan et al. [37]. The eluents used for separation were 200 mM NaOH, 500 mM NaOH, 500 mM NaOAc, and ultrapure water.

Separation was performed at a temperature of 30 °C on both columns, while detection was performed at 25 °C.

For the quantification of FODMAPs, extra-pure 50% sodium hydroxide solution (in water) (Fisher Chemical™, Brussels, Belgium), sodium acetate (99%+, NaOAc) (Thermo Scientific, Sunnyvale, USA), and standards were used. The standards included the following: fructose, galactose, glucose, sucrose, mannitol, sorbitol, 1-Kestose, nystose, stachyose hydrate (Sigma-Aldrich, Darmstadt, Germany), rhamnose monohydrate, xylitol, raffinose pentahydrate (Roth, Germany), lactose monohydrate, melibiose, maltitol (Thermo Scientific, Sunnyvale, CA, USA), verbascose, and 1,1,1-kestopentaose (Megazyme, Bray, Republic of Ireland). All carbohydrate reference standards were of >98% purity, except for 1,1,1-kestopentaose (which had a purity of 80%). Ultrapure water with a resistivity of 18.2 MΩ·cm and a total organic carbon (TOC) content of < 5 ppb (ASTM Type I) was used for the preparation of HPAEC-PAD eluents, all standard solutions, and samples; it was obtained from an Adrona Crystal pure water purification system (Riga, Latvia).

### 2.5. Total Fiber Content

The total fiber content in the bread samples was determined following the official AOAC 991.43 method [38].

### 2.6. Texture and Volume Measurements

The texture properties of bread were quantitatively assessed using a TA.XTplus Texture Analyzer (Stable Micro Systems, Surrey, UK) equipped with a 36 mm flat-end compression disc (probe P/36R). Firmness and resilience were determined following the AACC method 74-10.02 [39].

The volume of bread loaves was measured using a VolScan VSP 600 laser-based profiler (Stable Micro System, Godalming, England, UK). This method is approved with the AACCI Standard Method 10-16.01 [40].

### 2.7. Sensory Analyses

Sensory analyses of the prepared bread samples were performed by a panel of six highly trained assessors (three male and three female), aged 22–55 years, specifically trained in the evaluation of bread. The training included exercises in the identification of sensory descriptors and the evaluation of the intensity of sensory attributes. To ensure objectivity in obtaining the results, analyses were carried out in accordance with SRPS EN ISO 8589:2015 [41] and SRPS ISO 6658:2018 [42]. Testing took place in a sensory laboratory equipped with all the necessary facilities, under artificial light (1100 lx) and temperature control (22 °C). The panelists were asked to provide scores for seven parameters, namely the appearance, crust color, porosity, aroma, taste, odor, and aftertaste, expressing the intensity of each attribute on a nine-point hedonic scale (9—extremely good; 1—extremely bad). The samples were evaluated 24 h after baking. Each panelist received whole bread labeled with a randomly chosen three-digit number, and during their analysis, they were instructed to cut the samples in half and then into 10 mm thick slices. Drinking water was provided for palate cleansing between each sample.

Descriptions for parameters were the following: appearance—presence/absence of cracks on the surface of the sample and roughness/evenness of the surface; crust color—intensity of color; porosity—size of the holes in the crumb and homogeneity of the pores; aroma—olfactory perceptions caused by volatile substances released from a product in the mouth via the posterior nares; taste—gustatory perceptions (salty, sweet, sour, bitter) caused by soluble substances in the mouth; odor—detected when volatiles enter the nasal passage, as perceived by the olfactory system (volatiles are inhaled through the nose); aftertaste—gustatory—olfactory sensation left in the mouth after bread has been swallowed or spat out (persistence of flavor after the stimulating agent has gone), perceived as a positive characteristic.

### 2.8. Statistical Analyses

The experiments were performed in triplicate. Values are expressed as means ± standard deviation. The statistical calculations were performed with MS Excel 2010.

## 3. Results and Discussion

### 3.1. FODMAP Compound Reduction During Breadmaking

Our approach to the successfully created low-FODMAP wholegrain wheat and low-FODMAP wholegrain rye breads stemmed from one of the important goals, namely that the devised solution must not change the technological process of conventional (standard, traditional) bread production and its cost efficiency, because that would only slow down its introduction to the industrial production of low-FODMAP bread.

The experiments were designed in a way that would completely encompass the optimal conditions for the activation of available FODMAP-degrading enzymes in baker’s yeast (Figure 1).

A standard yeast bread formulation was used as a control and starting point for further optimization of both wholegrain wheat and wholegrain rye bread production (Table 1). During the experiments, the process parameters that varied included the dough temperature, baker’s yeast content, pH value of dough, duration of dough fermentation, and addition of baking powder.

Having studied the optimal conditions for the activity of the enzymes produced by baker’s yeast that break down FODMAP compounds, a certain temperature and pH value were chosen, optimizing the duration of the technological process, which resulted in a shorter production process compared to the conventional one.

The FODMAP compound contents determined in the produced bread samples are presented in Table 2.

First of all, the higher fermentation temperature of 40 °C was applied to bring the dough temperature closer to the optimal value for enzyme activity, but without initiating the denaturing of the enzyme complex and structural proteins of the dough. Dough fermentation at 40 °C led to 32% and 42% decreases in the FODMAP content of the wholegrain wheat and wholegrain rye bread samples, respectively. Especially significant was the decrease in GOS content, which could be due to the optimal temperature of 40 °C for the activity of α-galactosidase.

To investigate the influence of the baker’s yeast, the content added to the dough was first increased to 4%, and experiments were run at two dough fermentation temperatures (30 and 40 °C). A more intense degradation of FODMAP compounds was observed, resulting in further decreases of 41 and 30% for the wholegrain wheat and wholegrain rye bread samples, respectively.

The second major change made during optimization was the shortening of the dough fermentation time to 60 min. Considering the production cost efficiency, and also the nature of the substrate and presence of the yeast and endogenous enzymes in wholegrain flour, we optimized the process such that the dough fermentation process lasted 60 min (the fermentation in the dough mass will last 60 min). These experiments are justified based on the research of Struyf et al. [21], in which it was determined that in the presence of a large excess of yeast in the dough, all fructans can be broken down within 60 min of fermentation at a temperature of 30 °C. In our research, by gradually increasing the amount of yeast, we achieved satisfactory results, with 6% of fresh yeast calculated for the amount of flour.

Based on the research by Struyf et al. [30], during the fermentation of dough from wholegrain flour, it is also possible to accelerate FODMAP degradation by increasing the amount of substrate. As the products of yeast metabolism strongly influence the dough matrix, the yeast concentration and fermentation time are crucial factors for decreasing the pH value of dough via the production of acidic compounds. Furthermore, increasing the amounts of *S. cerevisiae* resulted in lower pH values in the dough. The authors have suggested that the decrease in pH value during fermentation enhanced protease activity and therefore weakened the gluten structure [16].

Since a pH of 4.4 is optimal for protease to degrade the protein network in the dough, it is important to shorten the fermentation process so that the pH value does not drop from 5 to 4.4 due to the significantly increased amount of yeast and the increased content of degradation products [16]. Furthermore, to prevent the pH dropping to 4.4, baking powder (BP) was added as the third major change performed during optimization. The addition of BP was also intended to create an optimal pH range for aldolase activity [28] to hydrolase the excess fructose generated via the addition of more yeast to the dough. Additionally, the BP neutralized the acidic notes in the flavor of the bread (generated via degradation during fermentation, mainly of organic acids). However, during dough fermentation at 30 °C for wholegrain wheat bread, a 60 min holding period was not sufficient to degrade the products that accumulated due to the depolymerization of inulin and FOSs. As a result, the combination of these process conditions represents an exception regarding the targeted reduction in the total content of FODMAP compounds. Conducting the process at 40 °C ensured that the fermentation in the dough mass took place at 37 °C, which is optimal for enzyme activity, and resulted in all bread samples having low FODMAP contents.

**Figure 2:**
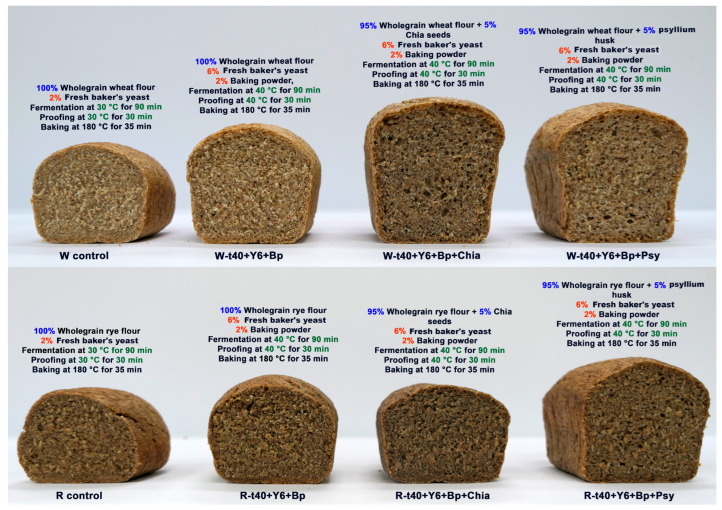
Cross-section of control and low-FODMAP bread loaves.

**Figure 3:**
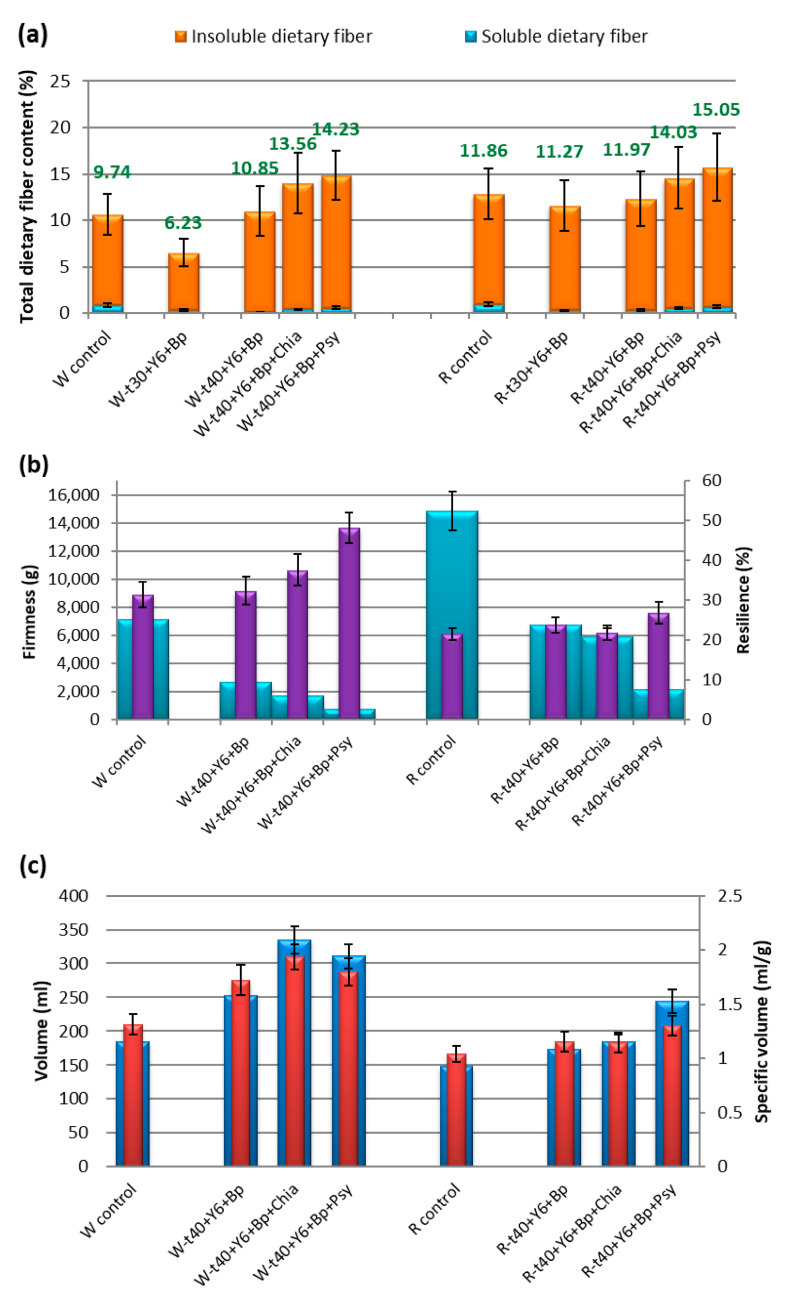
Physico-chemical properties of produced control and low-FODMAP wholegrain breads. (**a**) Total dietary fiber content, (**b**) Textural properties of bread (firmness and resilience), (**c**) Volume of bread loaves (volume and specific volume). Sample symbols are explained in Abbreviations section.

The FODMAP content measured in the samples with the addition of BP was higher compared to the samples without BP, but the volumes of loaves were higher by 38% and 16% compared to the control sample for wholegrain wheat and rye bread, respectively. This had a favorable effect on density, resulting in less compacted bread loaves with a lower firmness and a higher elasticity (resilience), consequently improving their texture and sensory properties (Figure 2 and Figure 3b). The most prominent improvement was in terms of the bread’s taste.

At pH 6, in the doughs with added BP, optimal conditions for the action of invertase, aldolase, and inulinase are created, but this specific dough pH value is further away from the optimal pH for the activity of alpha-galactosidase (pH 4). However, the dough pH is still in the appropriate range for maximum α-galactosidase activity (from 2 to 7.5) [44]. In the samples with addition of BP, FOS compounds were degraded to a lesser extent compared to the samples without the addition of BP, and the degree of degradation was higher, i.e., a lower FOS compound content. Aldolase removes excess fructose in contrast to glucose, and inulinase breaks down FODMAP compounds with DP > 5 and probably increases FOS content. At the same time, invertase does not have enough time to hydrolyze them.

### 3.2. Physico-Chemical Properties of the Produced Low-FODMAP Wholegrain Breads

A significant increase in TDF (mainly IDF) content was observed in all produced low-FODMAP breads in comparison to control samples (Figure 3a). This resulted in an unexpectedly lower firmness and an increase in total and specific bread volumes (Figure 3c), which is contradictory to the behavior and influence of IDF according to the latest research [45].

One of the major steps in the optimization process is the addition of baking powder, which until now, has never been combined with baker’s yeast in the bread industry. However, by adding the baking powder, we further activated yeast enzymes that hydrolyze FOS and GOS compounds and inulin (FODMAP inulin fractions), ensuring the production of sufficient amounts of CO_2_ for the bread to have a larger volume (Figure 3c) and to neutralize its acidity. This ultimately led to achieving a previously unattainable optimal bread sensory quality.

### 3.3. Sensory Properties of Bread Samples

Although the main goal of reducing the FODMAP content was achieved, the fourth major change implemented during optimization was the addition of grounded chia seeds or grounded psyllium husk to additionally increase the content of TDF and further enhance the sensory properties of the produced breads. Based on extensive preliminary experiments, the final choices as additional ingredients for low-FODMAP breads were chia seed gel and dry, ground psyllium husk. We therefore not only achieved sensory properties equal to the control breads but also exceeded them, thus obtaining outstanding and appealing products overall. The overall sensory impression, taste, smell, and aftertaste scores were the highest for the wholegrain wheat chia gel low-FODMAP bread and wholegrain rye psyllium husk low-FODMAP bread samples fermented at 40 °C.

**Figure 4:**
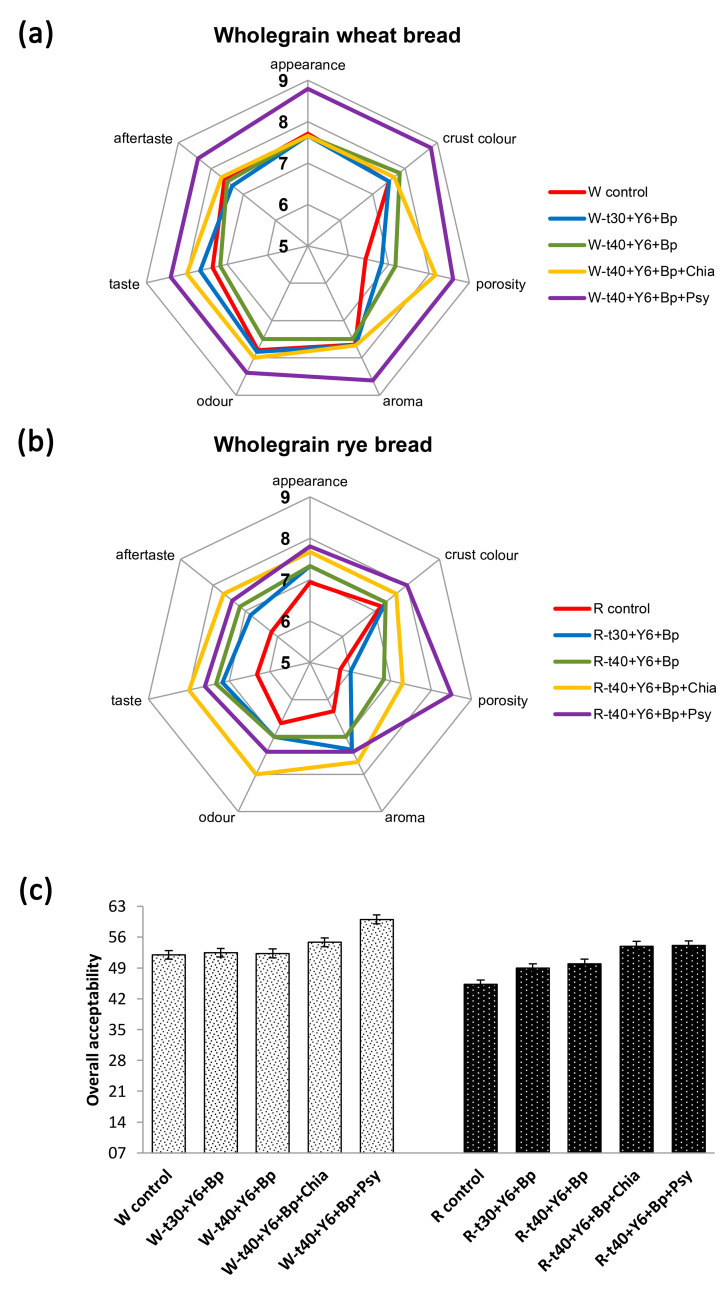
Results of sensory properties evaluation. (**a**) scores of sensory evaluation of wholegrain wheat bread, (**b**) scores of sensory evaluation of wholegrain rye bread, (**c**) overall acceptability score for wholegrain wheat and rye breads. Sample symbols are explained in Abbreviations section.

An expert sensory panel evaluated the produced breads and determined that they did not differ in taste and smell from the control breads (breads with standardized quality), with some even achieving better scores. In general, wholegrain wheat breads had a higher overall acceptability score, while the reduced FODMAP content in wholegrain rye bread had a higher impact on overall acceptability (Figure 4a–c).

## 4. Conclusions

The results presented in this study were acquired by optimizing the standard technological process of breadmaking via the addition of a chemical raising agent, baking powder, and baker’s yeast (the commonly added biological leavening agent). To the authors’ knowledge, this is the first time this combination of raising agents had been applied in breadmaking to reduce FODMAP contents. Additionally, the process of making these special types of wholegrain breads was shortened in comparison to the conventional process, thus making it more cost-effective. All of this resulted in reductions of 81 and 78% in the total oligosaccharides content in wholegrain wheat and wholegrain rye bread, respectively. Further, the addition of chia seed gel led to an almost 100% reduction in total oligosaccharides content. Low-FODMAP wholegrain wheat and rye breads also exhibited an increased TDF content, better texture properties, and a higher volume and specific volume. The produced low-FODMAP breads did not differ in taste and smell from the control breads (breads with standardized quality), while some even achieved better scores. In this regard, the low-FODMAP wholegrain wheat breads had a better taste, odor, and porosity in comparison to the control samples. The low-FODMAP wholegrain rye breads were assigned better scores for every evaluation parameter in comparison to the control samples. The enhancement in all evaluation parameters was more prominent in low-FODMAP wholegrain rye breads, although higher overall acceptability scores were achieved in low-FODMAP wholegrain wheat breads. Producing the above-mentioned bakery products does not require changes to the conventional bread-making process, and the bakery industry does not need to create special products for the population on a low-FODMAP diet. These products represent a common solution for staple high-fiber, low-FODMAP foods for the population with gastrointestinal disorders and for the healthy population, without compromising on nutritive quality, safety, and taste.

## Figures and Tables

**Table 1:** Procedure for breadmaking with wholegrain wheat or rye flour.

Ingredients		%Based on Flour						
	Wholegrain wheat flour	100	100		100		95	95
	Water	55	55		55		80	100
	Baker’s yeast (*S. cerevisiae*)	2	6		6		6	6
	Salt	1.5	1.8		1.8		1.8	1.8
	Sunflower oil	0	0		2		2	2
	Baking powder	0	0		2		2	2
	Sucrose	0.5	1.5		1.5		1.5	1.5
	Ground chia seeds	0	0		0		5	0
	Ground psylium husk	0	0		0		0	5
**Conditions**								
	Temperature (°C)	30	30	40	30	40	40	
	Rest time after kneeding (min)	90	60		60		60	
	Rest time after molding (min)	30	30		30		30	
	Relative humidity during resting (%)	90	90		90		90	
**Ingredients**		**%Based on Flour**						
	Wholegrain rye flour	100	100		100		95	95
	Water	63	63		63		83	115
	Baker’s yeast (*S. cerevisiae*)	2	6		6		6	6
	Salt	1.5	1.8		1.8		1.8	1.8
	Sunflower oil	0	0		2		2	2
	Baking powder	0	0		2		2	2
	Sucrose	0.5	1.5		1.5		1.5	1.5
	Ground chia seeds	0	0		0		5	0
	Groudn psylium husk	0	0		0		0	5
**Conditions**								
	Temperature (°C)	30	30	40	30	40	40	
	Rest time after kneeding (min)	90	60		60		60	
	Rest time after molding (min)	30	30		30		30	
	Relative humidity during resting (%)	90	90		90		90	

**Table 2:** FODMAPs content in breads produced from wholegrain wheat and wholegrain rye flours.

Wholegrain Wheat					
	FOS (in Range) g/100 g “as is”	GOS (in Range) g/100 g “as is”	FOS + GOS (in Range) g/100 g “as is”	Fructose in Excess to Glucose (in Range) g/100 g “as is”	Total Polyols (in Range) g/100 g “as is”
**Benchmark values for different FODMAPs groups** [43]			**Total oligosaccharides** (FOS + GOS) **0.6**	**0.3**	**0.8**
**W control**	1.626 ± 0.608	0.082 ± 0.082	1.708 ± 0.690	0.036 ± 0.008	0.170 ± 0.011
**W-t30+Y6**	0.574 ± 0.313	0.052 ± 0.052	0.615 ± 0.273	0.000 ± 0.000	0.107 ± 0.068
**W-t30+Y6+Bp**	1.213 ± 0.695	0.073 ± 0.073	1.213 ± 0.695	0.001 ± 0.001	0.107 ± 0.098
**W-t40+Y6**	0.252 ± 0.252	0.061 ± 0.061	0.301 ± 0.204	0.008 ± 0.008	0.151 ± 0.091
**W-t40+Y6+Bp**	0.229 ± 0.174	0.115 ± 0.015	0.339 ± 0.183	0.001 ± 0.001	0.133 ± 0.077
**W-0+Y6+Bp+Chia**	0.000 ± 0.000	0.034 ± 0.034	0.034 ± 0.034	0.002 ± 0.002	0.090 ± 0.029
**W-t40+Y6+Bp+Psy**	0.014 ± 0.006	0.290 ± 0.262	0.303 ± 0.268	0.001 ± 0.001	0.000 ± 0.000
**Wholegrain Rye**					
	**FOS** **(in Range)** **g/100 g “as is”**	**GOS** **(in Range)** **g/100 g “as is”**	**FOS + GOS** **(in Range)** **g/100 g “as is”**	**Fructose in Excess to Glucose** **(in Range)** **g/100 g “as is”**	**Total Polyols (in Range)** **g/100 g “as is”**
**Benchmark values for different FODMAPs groups** [43]			**Total oligosaccharides** (FOS + GOS) **0.6**	**0.3**	**0.8**
**R control**	0.876 ± 0.530	0.621 ± 0.535	1.497 ± 0.006	0.142 ± 0.004	0.187 ± 0.002
**R-t30+Y6**	0.132 ± 0.106	0.047 ± 0.047	0.177 ± 0.061	0.186 ± 0.021	0.140 ± 0.063
**R-t30+Y6+Bp**	0.039 ± 0.037	0.046 ± 0.044	0.164 ± 0.043	0.131 ± 0.076	0.109 ± 0.045
**R-t40+Y6**	0.128 ± 0.118	0.043 ± 0.043	0.166 ± 0.080	0.005 ± 0.005	0.113 ± 0.045
**R-t40+Y6+Bp**	0.071 ± 0.031	0.249 ± 0.247	0.340 ± 0.237	0.009 ± 0.007	0.054 ± 0.044
**R-40+Y6+Bp+Chia**	0.024 ± 0.023	0.031 ± 0.031	0.058 ± 0.050	0.001 ± 0.001	0.114 ± 0.033
**R-t40+Y6+Bp+Psy**	0.126 ± 0.051	0.429 ± 0.017	0.554 ± 0.033	0.044 ± 0.044	0.000 ± 0.000

values marked with bold exceed the benchmark values for Low-FODMAP products per compound category. FOS—fructooligosaccharides, GOS—galactooligosaccharide, only total polyols were presented since the individual values did not exceed the benchmark value.

## Data Availability

The data presented in this study are available on request from the corresponding author.

## Abbreviations

**W control**	wholegrain wheat bread produced by 90 min dough fermentation (100% wholegrain wheat flour) at 30 °C with the addition of 2% of baker’s yeast;
**W-t30+Y6**	wholegrain wheat bread produced by 60 min dough fermentation (100% wholegrain wheat flour) at 30 °C with the addition of 6% of baker’s yeast;
**W-t40+Y6**	wholegrain wheat bread produced by dough fermentation (100% wholegrain wheat flour) for 60 min at 40 °C with the addition of 6% of baker’s yeast
**W-t30+Y6+Bp**	wholegrain wheat bread produced by 60 min dough fermentation (100% wholegrain wheat flour) at 30 °C with the addition of 6% of baker’s yeast and 2% of baking powder;
**W-t40+Y6+Bp**	wholegrain wheat bread produced by dough fermentation (100% wholegrain wheat flour) for 60 min at 40 °C with the addition of 6% of baker’s yeast and 2% of baking powder;
**W-t40+Y6+Bp+Chia**	wholegrain wheat bread produced by 60 min dough fermentation (95% wholegrain wheat flour + 5% of Chia seeds) at 40 °C with the addition of 6% of baker’s yeast and 2% of baking powder;
**W-t40+Y6+Bp+Psy**	wholegrain wheat bread produced by 60 min dough fermentation (95% wholegrain wheat flour + 5% of Psylium husk) at 40 °C with the addition of 6% of baker’s yeast and 2% of baking powder;
**R control**	wholegrain rye bread produced by 90 min dough fermentation (100% wholegrain rye flour) at 30 °C with the addition of 2% of baker’s yeast;
**R-t30+Y6**	wholegrain rye bread produced by 60 min dough fermentation (100% wholegrain rye flour) at 30 °C with the addition of 6% of baker’s yeast;
**R-t40+Y6**	wholegrain rye bread produced by dough fermentation (100% wholegrain rye flour) for 60 min at 40 °C with the addition of 6% of baker’s yeast;
**R-t30+Y6+Bp**	wholegrain rye bread produced by 60 min dough fermentation (100% wholegrain rye flour) at 30 °C with the addition of 6% of baker’s yeast and 2% of baking powder;
**R-t40+Y6+Bp**	wholegrain rye bread produced by dough fermentation (100% wholegrain rye flour) for 60 min at 40 °C with the addition of 6% of baker’s yeast and 2% of baking powder;
**R-t40+Y6+Bp+Chia**	wholegrain rye bread produced by 60 min dough fermentation (95% wholegrain rye flour + 5% of Chia seeds) at 40 °C with the addition of 6% of baker’s yeast and 2% of baking powder;
**R-t40+Y6+Bp+Psy**	wholegrain rye bread produced by 60 min dough fermentation (95% wholegrain rye flour + 5% of Psylium husk) at 40 °C with the addition of 6% of baker’s yeast and 2% of baking powder
